# Dosimetric risk factors for therapy‐related pneumonitis in patients with immunotherapy plus chemotherapy concurrent thoracic radiotherapy

**DOI:** 10.1002/pro6.70102

**Published:** 2026-11-03

**Authors:** Ying Lu, Youjia Wang, Guang Han, Jianping Bi, Guoliang Pi, Xinbin Pan, Vivek Verma, Xiaofang Dai, Jiangqiong Huang, Ying Li

**Affiliations:** ^1^ Department of Radiation Oncology Hubei Cancer Hospital, Tongji Medical College Huazhong University of Science and Technology Wuhan China; ^2^ Department of Radiation Oncology Guangxi Medical University Cancer Hospital Nanning China; ^3^ Department of Radiation Oncology The University of Texas MD Anderson Cancer Center Houston Texas USA; ^4^ Cancer Center Union Hospital, Tongji Medical College, Huazhong University of Science and Technology Wuhan China; ^5^ Radiation Oncology Institute Union Hospital, Tongji Medical College, Huazhong University of Science and Technology Wuhan China

**Keywords:** Chemoradiotherapy, Esophageal cancer, Immune checkpoint inhibitor, Lung cancer, Pneumonitis

## Abstract

**Objective:**

Combining immune checkpoint inhibitors (ICIs) with chemoradiotherapy is being actively investigated to improve clinical outcomes; however, this strategy may also increase the risk of treatment‐related toxicities. This multicenter study aimed to identify clinical and dosimetric predictors of treatment‐related pneumonitis (TRP) in this patient population, focusing on modifiable predictors of toxicity.

**Methods:**

A retrospective review was conducted on 69 patients with lung or esophageal cancer treated at three hospitals. All patients received concurrent ICIs, chemotherapy, and thoracic radiotherapy at a dose of 50–60 Gy (in 2 Gy fractions) using inverse‐planned techniques. TRP was graded according to the Common Terminology Criteria for Adverse Events version 5.0. Logistic regression modeling and receiver operating characteristic (ROC) analysis were employed for statistical evaluation.

**Results:**

Any‐grade TRP occurred in 56.5% of patients. Grade 2–5 TRP occurred in 30.4%, 8.7%, 1.4%, and 0% of patients, respectively. Age (*p *= 0.01; *OR *= 1.1) and mean lung dose (MLD) (*p *= 0.02; *OR *= 2.4) independently contributed to the risk of any‐grade TRP. Age (*p *= 0.03; *OR *= 1.1) and MLD (*p *= 0.04; *OR *= 1.7) were also identified as risk factors for grade 2+ TRP. Other parameters, such as lung *V*5, *V*10, *V*15, *V*20, and *V*30 were not significantly associated with TRP. ROC analysis identified 13.6 Gy as the optimal MLD threshold for predicting both any‐grade and grade 2+ TRP.

**Conclusions:**

MLD and age were associated with a higher risk of TRP in patients receiving concurrent chemoradiotherapy and ICIs. These findings may help inform revisions to lung dose constraints, although validation in large‐scale prospective trials is warranted.

## Graphical abstract

In this study, mean lung dose (MLD) and age were associated with an increased risk of treatment‐related pneumonitis in patients receiving concurrent chemoradiotherapy and immunotherapy, providing a reference for revising lung dose constraints.

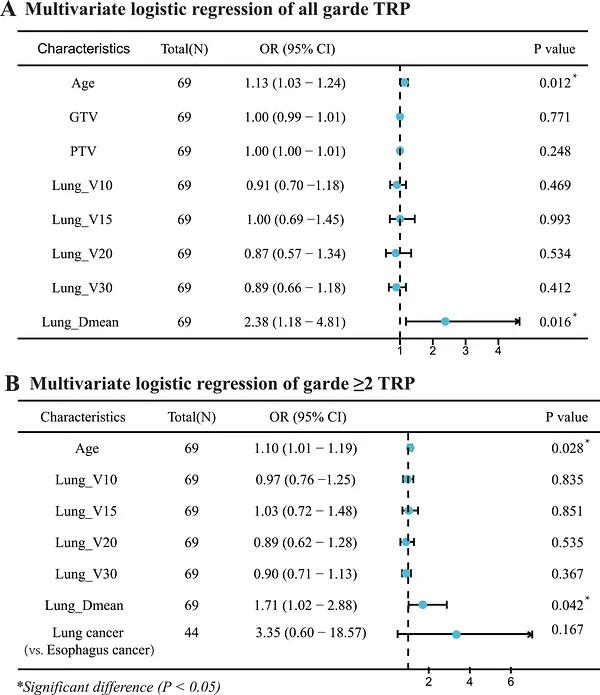

## 1 INTRODUCTION

Concurrent chemoradiotherapy (CCRT) is the standard treatment for inoperable intrathoracic tumors. Numerous studies and clinical trials have indicated that thoracic radiotherapy (TRT) enhances antitumor immune responses, and its combination with immunotherapy may produce synergistic effects that improve clinical outcomes.^1^, ^2^

CCRT followed by durvalumab is the standard treatment for locally advanced non‐small cell lung cancer (LA‐NSCLC).^3^ The PALACE‐1 study indicates that pembrolizumab combined with radiotherapy and chemotherapy may benefit patients with esophageal squamous cell carcinoma (ESCC).^4^ However, some patients are unable to receive durvalumab after CCRT because of adverse events or poor performance status.^5^, ^6^

Preclinical models suggest that concurrent administration of ICIs and radiotherapy may enhance therapeutic efficacy.^2^, ^7^ Integrating ICIs with Chemoradiotherapy could extend these benefits to patients eligible for CCRT and potentially improve outcomes in a broader patient population.^7^ This concurrent trimodal therapeutic approach is currently being investigated for unresectable stage III NSCLC and ESCC.^8^, ^9^, ^10^, ^11^

Although theoretically beneficial, combining radiotherapy with immunotherapy may increase the risk of pulmonary toxicity. Because immune‐ and radiation‐related pneumonitis share overlapping pathways in their onset and progression, determining whether pneumonitis is attributable to immunotherapy or radiotherapy during combined treatment remains challenging.^12^, ^13^ Consequently, in clinical practice, pneumonitis arising in this context is often termed treatment‐related pneumonitis (TRP).

Although TRP may be modifiable through established radiation dose–volume parameters, whether these parameters apply to, or require adjustment for, the setting of concurrent immune checkpoint inhibitors (ICIs) remains unknown. In this study, we conducted a real‐world multicenter investigation to evaluate the efficacy of concurrent trimodal treatment with ICIs, chemotherapy, and thoracic radiotherapy. We also evaluated dosimetric parameters as predictors of TRP to support the development of dosimetric constraints for patients undergoing concurrent immunotherapy and CCRT.

## 2 MATERIALS AND METHODS

### 2.1 Patients and treatment

This retrospective multicenter study enrolled patients with lung cancer (LC) or ESCC who received concurrent thoracic radiotherapy, chemotherapy, and ICIs at Hubei Cancer Hospital, Wuhan Union Hospital, or Guangxi Cancer Hospital between July 21, 2020, and October 1, 2023.

All patients were treated with intensity‐modulated radiation therapy (IMRT) or volumetric‐modulated arc radiotherapy (VMAT) to a total dose of 50–60 Gy in 2‐Gy fractions. Radiation therapy (RT) was planned and delivered according to standardized institutional protocols and established dose constraints, including those recommended by QUANTEC guidelines.^14^ This study was approved by the Ethics Committee of Hubei Cancer Hospital (Approval Number: LLHBCH2024YN‐047), and obtained informed consent and recognition from all participating sub‐centers.

### 2.2 Data collection

Eligibility required: (1) a pathological diagnosis of LC or ESCC, with staging supported by computed tomography (CT) or positron emission tomography‐computed tomography (PET‐CT); (2) receipt of CCRT combined with ICIs; and (3) available follow‐up data, with clinical assessments performed at least every 3 months or more frequently as clinically indicated until disease progression. Patients were excluded if medical records were missing or if pre‐radiotherapy imaging or hematological examination data were unavailable.

Demographic characteristics, clinical findings, laboratory test results, imaging data, and treatment details were retrospectively extracted from patient medical records. Radiation‐related variables were also collected, including target volumes and organs‐at‐risk parameters: gross tumor volume (GTV), planning target volume (PTV), lung volumes receiving at least 5, 10, 15, 20, and 30 Gy (*V*5, *V*10, *V*15, *V*20, and *V*30), and mean lung dose (MLD).

### 2.3 Toxicity assessments

Treatment‐related adverse events were graded according to the Common Terminology Criteria for Adverse Events (CTCAE) version 5.0. Suspected TRP was independently reviewed and graded by at least one radiologist and one radiation oncologist after alternative etiologies, including infection and disease progression, had been excluded. For this analysis, acute and late‐onset TRP were combined as a composite endpoint, without further temporal stratification.

### 2.4 Statistical analysis

Categorical variables were compared using the χ^2^ test, and continuous variables were compared using the Mann–Whitney *U* test. Univariate and multivariate logistic regression models were used to identify factors associated with TRP, and receiver operating characteristic (ROC) analysis was used to assess predictive performance. Variables with *p *< 0.20 in univariate analyses were entered into multivariate analyses. Statistical analyses and visualization were performed using R version 3.6.3 (R Foundation for Statistical Computing, Vienna, Austria), with two‐tailed *p* ≤ 0.05 considered statistically significant.

## 3 RESULTS

### 3.1 Patient characteristics

A total of 69 patients received concurrent trimodality therapy across the three participating institutions between July 21, 2020, and October 1, 2023. Baseline patient characteristics are summarized in Table 1. During thoracic radiotherapy, all patients received at least one cycle of chemotherapy and immunotherapy, comprising programmed death‐1 (PD‐1) inhibitors (59.4%), programmed death‐ligand 1 (PD‐L1) inhibitors (24.6%), or dual immunotherapy (15.9%). The cohort included patients with NSCLC (26.1%), SCLC (37.7%), and ESCC (36.2%).

**TABLE 1:** Basic characteristics of patients.

Characteristic	ALL patients
**Total**	69
**Age (mean (SD))**	62.46(8.01)
**Sex (%)**	
Female	8 (11.6)
Male	61 (88.4)
**Smoke (%)**	
No	15 (21.7)
Yes	54 (78.3)
**Tumor types (%)**	
NSCLC	18 (26.1)
SCLC	26 (37.7)
Esophagus cancer	25 (36.2)
**T stage (%)**	
T1–2	16 (23.2)
T3–4	53 (76.8)
**N stage (%)**	
N0‐1	11 (18.0)
N2	35 (57.4)
N3	15 (24.6)
**Stage (%)**	
II	6 (8.7)
III	63 (91.3)
**Performance status (%)** ^a^	
ECOG 0	28 (40.6)
ECOG 1	41 (59.4)
**Immunotherapy (%)**	
PD‐1	41 (59.4)
PD‐L1	17 (24.6)
Dual immunotherapy	11 (15.9)
**Chemo cycle (%)**	
<3	41 (59.4)
≥4	28 (40.6)
**PD‐L1 expression (%)** ^b^	
<1%	2 (2.9)
≥1%	4 (5.8)
Unknown	63 (91.3)
**Genetic testing (%)**	
EGFR E19(+)	2 (2.9)
EGFR(‐)	4 (5.8)
PIK3CA(+)	1 (1.4)
ALK(‐)	4 (5.8)
Unknown	60 (87.0)

Abbreviations: Chemo cycle, chemotherapy cycle; dual immunotherapy, combined application of two immune checkpoint inhibitors; ECOG, Eastern Cooperative Oncology Group; PD‐1, programmed death 1; PD‐L1, programmed death ligand 1.

^a^ Scored on a scale of 0 to 5, with 0 and 1 indicating fully active and restricted but ambulatory, respectively, and 5 indicating dead.

^b^ Scored on a scale of 1% to 100%, indicating the percentage of viable tumor cells on staining.

Radiotherapy‐related characteristics of the study cohort are summarized in Table 2. The median GTV and PTV were 62.0 cc [interquartile range (IQR) 37.4‐108.6] and 390.7 cc [IQR 254.8–485.5], respectively. Median lung dosimetry values were *V*5 49.3% (IQR 39.7–56.8), *V*10 36.1% (IQR 27.5–40.1), *V*15 28.42% (IQR 22.2‐31.4), *V*20 22.0% (IQR 17.0‐25.5), *V*30 14.0% (IQR 10.1–18.3), and MLD 12.5 Gy (IQR 9.9–14.7).

**TABLE 2:** Thoracic radiotherapy characteristics.

Dosimetric Parameters	median [IQR]
GTV	62.00 [37.39, 108.60]
PTV	390.70 [254.80, 485.50]
Lung V5	49.30 [39.67, 56.83]
Lung V10	36.09 [27.50, 40.09]
Lung V15	28.42 [22.20, 31.14]
Lung V20	21.95 [17.02, 25.50]
Lung V30	14.00 [10.10, 18.25]
Lung D_mean_	12.54 [9.94, 14.70]

Abbreviations: D_mean_, mean lung dose; GTV, gross tumor volume; IQR, interquartile range; Lung *V*5, volume of lungs receiving ≥5 Gy; Lung *V*10, volume of lungs receiving ≥10 Gy; Lung *V*15, volume of lungs receiving ≥15 Gy; Lung *V*20, volume of lungs receiving≥20 Gy; Lung *V*30, volume of lungs receiving ≥30 Gy; PTV, planning target volume.

### 3.2 TRP

The median follow‐up duration after thoracic irradiation was 30.4 months (IQR, 22.9–38.0 months). The overall incidence of any‐grade TRP was 56.5% (39/69), with grade‐specific incidences as follows: grade 1, 15.9% (11/69), grade 2, 30.4% (21/69), grade 3, 8.7% (6/69), grade 4, 1.4% (1/69), and no cases of fatal (grade 5) TRP observed. Among the 39 patients who developed TRP, the median interval from initiation of thoracic RT to TRP onset was 17.9 weeks (IQR 10.9–22.9), and the median TRP duration was 16.7 weeks (IQR 9.9‐30.3). Table 3 summarizes the characteristics of the seven patients who developed grade ≥3 TRP.

**TABLE 3:** Individual characteristics of the seven patients with grade ≥3 TRP.

Age	ECOG	Smoking	Pathology	Immunotherapy	Chemo cycle	*V*5 (%)	*V*10 (%)	*V*15 (%)	*V*20 (%)	*V*30 (%)	MLD (Gy)	symptoms
65	1	Yes	NSCLC	PD‐1	≥4	60.42	41.20	31.00	24.63	19.42	15.66	dyspnea, wheezing, fever, cough
70	0	Yes	NSCLC	Dual immunotherapy	≥4	24.73	19.80	17.60	14.96	7.71	7.47	wheezing, fever, cough
73	1	Yes	NSCLC	Dual immunotherapy	<3	61.60	48.90	37.90	31.07	28.80	17.66	cough
72	1	Yes	EC	PD‐1	<3	43.70	34.10	25.30	16.40	5.96	9.44	wheezing, cough
76	1	Yes	EC	PD‐1	<3	50.00	33.01	17.00	16.14	1.50	8.00	hemoptysis, cough
62	0	Yes	SCLC	PD‐L1	<3	29.00	23.97	21.24	19.00	14.00	9.94	cough
63	1	Yes	SCLC	PD‐L1	<3	51.00	35.30	28.69	23.00	17.00	16.06	fever, cough

Abbreviations: Chemo cycle, chemotherapy cycle; Dual immunotherapy, Combined application of two immune checkpoint inhibitors; EC, esophageal cancer; ECOG, Eastern Cooperative Oncology Group Performance Status; *V*5, volume of lungs receiving ≥5 Gy; Lung *V*10, volume of lungs receiving≥10 Gy; Lung *V*15, volume of lungs receiving ≥15 Gy; Lung *V*20, volume of lungs receiving ≥20 Gy; Lung *V*30, volume of lungs receiving ≥30 Gy; MLD, mean lung dose; NSCLC, non‐small cell lung cancer; PD‐1, programmed death 1; PD‐L1, programmed death ligand 1; SCLC, small cell lung cancer; TRP, treatment‐related pneumonitis.

### 3.3 Predictors of TRP

To identify factors associated with TRP, logistic regression analyses were performed for any‐grade TRP and for grade ≥2 TRP. As shown in Tables 4 and 5, age [odds ratio (*OR*) 1.1, 95% CI 1.0–1.2, *p *= 0.01] and MLD (*OR* 2.4, 95% CI 1.2–4.8, *p *= 0.02) were independent risk factors for any‐grade TRP. In the analysis of grade ≥2 TRP, age (*OR* 1.1, 95% CI 1.0–1.2, *p *= 0.03) and MLD (*OR* 1.7, 95% CI 1.0–2.9, *p *= 0.04) remained independent risk factors.

**TABLE 4:** Univariate and multivariate logistic analysis of any‐grade TRP.

		Univariate regression analysis		OR (95% CI)	
Characteristics	Total (*N*)	OR (95% CI)	*p*	OR (95% CI)	*p*
Age	69	1.06 (0.99–1.13)	0.089	1.13 (1.03–1.24)	0.012
GTV	69	1.01 (1.00–1.01)	0.091	1.00 (0.99–1.01)	0.771
PTV	69	1.01 (1.01–1.01)	0.039	1.00 (1.00–1.01)	0.248
Lung_*V*5	69	1.03 (0.99–1.07)	0.220		
Lung_*V*10	69	1.06 (1.00–1.12)	0.070	0.91 (0.70 –1.18)	0.469
Lung_*V*15	69	1.08 (1.00–1.17)	0.059	1.00 (0.69–1.45)	0.993
Lung_*V*20	69	1.10 (1.01–1.21)	0.049	0.87 (0.57–1.34)	0.534
Lung_*V*30	69	1.07 (0.99–1.16)	0.107	0.89 (0.66–1.18)	0.412
Lung_D_mean_	69	1.25 (1.05–1.47)	0.016	2.38 (1.18–4.81)	0.016
**Sex**					
Female	8	1.00 (Reference)			
Male	61	1.35 (0.31–5.89)	0.693		
**Smoking**					
No	15	1.00 (Reference)			
Yes	54	1.18 (0.37–3.72)	0.778		
**Pathology**					
Esophagus cancer	25	1.00 (Reference)			
Lung cancer	44	1.33 (0.50–3.58)	0.568		
**Immunotherapy**					
Dual immunotherapy	11	1.00 (Reference)			
PD‐1	41	0.81 (0.20–3.20)	0.760		
PD‐L1	17	0.51 (0.11–2.40)	0.393		
**Chemo_cycle**					
<3	41	1.00 (Reference)			
≥4	28	1.33 (0.50–3.54)	0.562		

Abbreviations: Chemo cycle, chemotherapy cycle; CI, confidence interval. N, number; GTV, gross tumor volume; IQR, interquartile range; Lung D_mean_, mean lung dose; Lung *V*5, volume of lungs receiving ≥5 Gy; Lung *V*10, volume of lungs receiving ≥10 Gy; Lung *V*15, volume of lungs receiving ≥15 Gy; Lung *V*20, volume of lungs receiving ≥20 Gy; Lung *V*30, volume of lungs receiving ≥30 Gy; OR, odds ratio; PTV, planning target volume; TRP, treatment‐related pneumonitis.

**TABLE 5:** Univariate and multivariate Logistic regression analysis of grade ≥2 TRP.

	Univariate regression analysis		Multivariate regression	
Characteristics	OR (95% CI)	*p*	OR (95% CI)	*p*
Age	1.04 (0.98–1.11)	0.190	1.10 (1.01–1.19)	0.028
GTV	1.00 (1.00–1.01)	0.222		
PTV	1.00 (1.00–1.00)	0.339		
Lung_*V*5	1.02 (0.98–1.06)	0.422		
Lung_*V*10	1.04 (0.98–1.11)	0.157	0.97 (0.76–1.25)	0.835
Lung_*V*15	1.07 (0.99–1.16)	0.107	1.03 (0.72–1.48)	0.851
Lung_*V*20	1.10 (0.99–1.22)	0.066	0.89 (0.62–1.28)	0.535
Lung_*V*30	1.08 (0.99–1.17)	0.088	0.90 (0.71–1.13)	0.367
Lung_*D*_mean_	1.22 (1.03–1.45)	0.021	1.71 (1.02–2.88)	0.042
**Sex**				
Female	1.00 (Reference)			
Male	0.65 (0.15–2.84)	0.566		
**Smoking**				
No	1.00 (Reference)			
Yes	0.73 (0.23–2.30)	0.588		
**Pathology**				
Esophagus cancer	1.00 (Reference)		1.00 (Reference)	
Lung cancer	2.35 (0.82–6.74)	0.113	3.35 (0.60–18.57)	0.167
**Immunotherapy**				
Dual immunotherapy	1.00 (Reference)			
PD‐1	0.77 (0.20–2.94)	0.700		
PD‐L1	0.84 (0.18–3.88)	0.823		
**Chemo_cycle**				
<3	1.00 (Reference)			
≥4	1.17 (0.44–3.11)	0.750		

Abbreviations: Chemo cycle: chemotherapy cycle; CI: confidence interval. N: number; GTV: gross tumor volume; IQR: interquartile range; Lung *D*_mean_: mean lung dose; Lung *V*10: volume of lungs receiving ≥10 Gy; Lung *V*15: volume of lungs receiving ≥15 Gy; Lung *V*20: volume of lungs receiving ≥20 Gy; Lung *V*30: volume of lungs receiving ≥30 Gy; Lung *V*5: volume of lungs receiving ≥5 Gy; OR: odds ratio; PTV: planning target volume; TRP: treatment‐related pneumonitis.

ROC analysis was performed to evaluate the predictive performance of the identified risk factors (Figures 1 and 2). For any‐grade TRP, the area under the curve (AUC) was 0.61 for age and 0.68 for MLD. For grade ≥2 TRP, the AUC values were 0.59 and 0.67 for age and MLD, respectively. To facilitate clinical application, optimal cutoff values for age and MLD were derived from the ROC curves. For any‐grade TRP, the optimal cutoffs were 59.5 years for age and 13.6 Gy for MLD. For grade ≥2 TRP, the corresponding optimal cutoffs were 68.5 years and 13.6 Gy.

**FIGURE 1:**
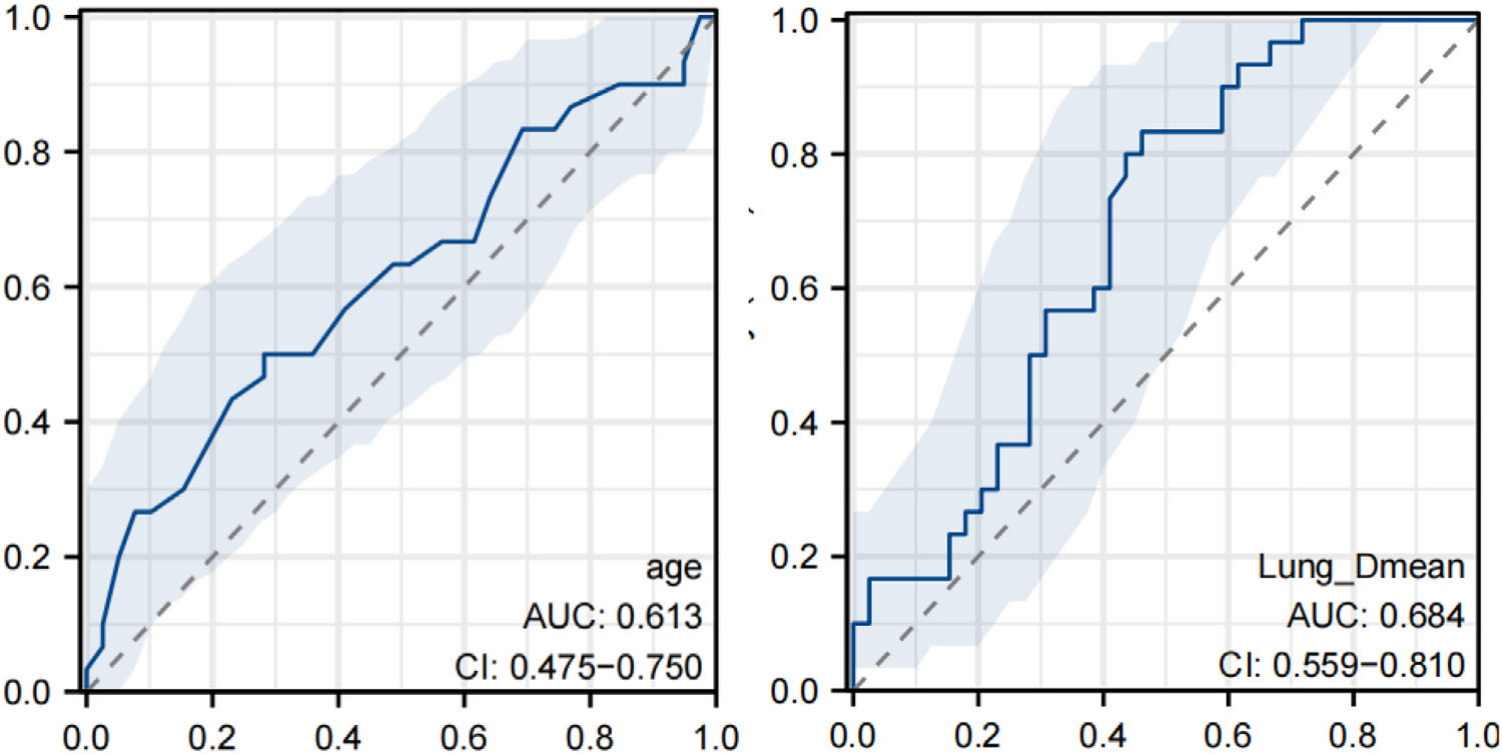
Receiver operating characteristic (ROC) curve of any‐grade TRP. TRP, treatment‐related pneumonitis; AUC, area under the curve; CI, confidence interval; Lung *D*_mean_, mean lung dose.

**FIGURE 2:**
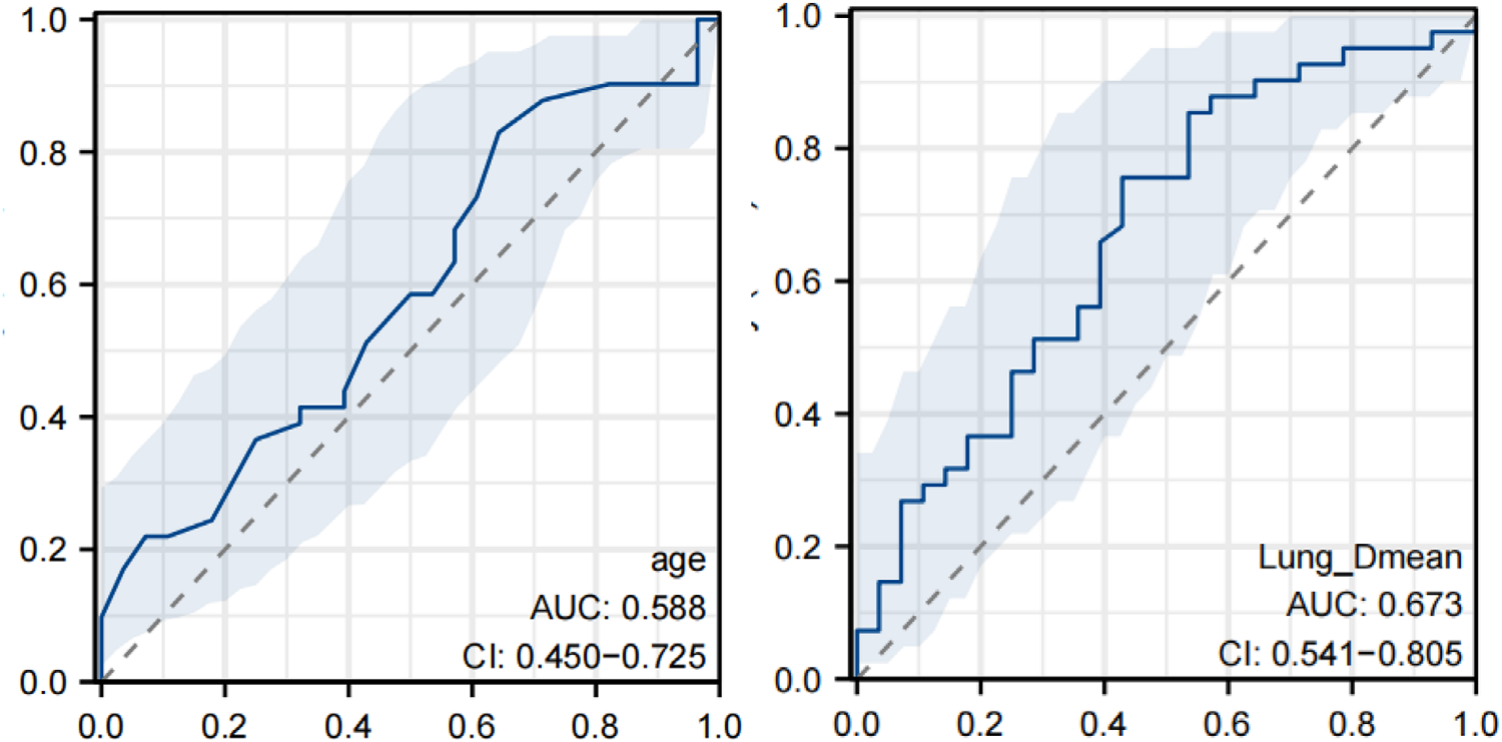
Receiver operating characteristic (ROC) curve of grade ≥2 TRP. TRP, treatment‐related pneumonitis; AUC, area under the curve; CI, confidence interval; Lung *D*_mean_, mean lung dose.

## 4 DISCUSSION

Although available data indicate that TRP occurs during trimodality therapy (CCRT + ICIs), the risk factors associated with its development have not, to our knowledge, been systematically examined. This study is therefore the first known multicenter analysis to evaluate factors associated with pulmonary toxicity in this treatment model. Radiation dosimetry, particularly MLD and *V*20^14^, is a strong predictor of radiation pneumonitis (RP). However, whether conventional thresholds for these metrics remain applicable in patients receiving concurrent trimodality treatment remains uncertain. The findings of this multicenter study suggest that MLD constraints based on QUANTEC standards may require reassessment when thoracic radiotherapy is administered concurrently with ICIs.

Our previous research demonstrated that, among patients previously exposed to immunotherapy who subsequently underwent thoracic radiotherapy, an MLD of 8.8 Gy predicted a 50% risk of any‐grade RP. We hypothesized that this threshold would differ in the concurrent ICI‐CCRT setting. In the present study, the MLD threshold was 13.6 Gy, which is notably higher than that observed in the prior sequential treatment setting.^15^, ^16^ This difference may reflect the lower cumulative number of ICI cycles typically delivered with concurrent therapy, whereas patients in earlier studies had received multiple ICI cycles before thoracic radiotherapy. Because the AUC for this threshold ranged from 0.67–0.68, 13.6 Gy should be interpreted as a preliminary estimate requiring evaluation in large‐scale prospective studies. Nonetheless, these findings collectively suggest that established QUANTEC dose constraints may need to be re‐evaluated in the era of immunotherapy.

The elevated risk of TRP observed in our concurrent‐therapy cohort may be attributed to the synergistic immunopathologic interactions between RT and ICIs. RT induces immunogenic cell death, promotes the release of tumor‐associated antigens and damage‐associated molecular patterns, and generates a pro‐inflammatory microenvironment enriched with cytokines, such as IL‐6 and TNF‐α. Concurrently, ICIs inhibit the PD‐1/PD‐L1 pathway, reactivate T cells, and may amplify the immune response initiated by RT.^17^ Excessive immune activation may then promote cytokine release and widespread infiltration of cytotoxic T cells, macrophages, and neutrophils into normal lung tissue, ultimately leading to TRP. Radiation recall pneumonitis further supports the persistence of immune memory and prolonged immune activation.^18^, ^19^ Consequently, the lung dose parameters identified in this study (e.g., MLD, and V20) may indirectly reflect the magnitude of the subsequent immune response, because larger irradiated volumes and higher doses may produce greater antigen release and a stronger synergistic inflammatory reaction, thereby increasing TRP risk.

The incidence of grade ≥2 TRP in our cohort was 40.6%, which is notably higher than the 16%–18% typically reported in the literature.^20^, ^21^ The incidence of grade ≥3 TRP was comparable to that reported in the NICOLAS trial (11.7%) but higher than the rates reported in KEYNOTE‐799 (8.0% in cohort A, and 6.9% in cohort B) and the DETERRED study (3.0%).^10^, ^20^, ^22^ This discrepancy may be partly attributable to differences in study populations. Notably, real‐world data and the DOLPHIN study in Japan also reported a high incidence of any‐grade pneumonitis in Asian populations following sequential ICI‐CCRT.^8^, ^23^ A meta‐analysis comparing the incidence of pneumonitis after CCRT between Asian and non‐Asian populations further supported this observation.^24^ Some researchers have suggested that Asian patients may be more susceptible to TRP because of lower lung radiation tolerance;^22^ however, further clinical data are needed to confirm this possibility.

Consistent with previous reports,^16^, ^23^, ^25^ our study identified age, in addition to MLD, as a factor associated with TRP. A meta‐analysis reported increased pneumonitis rates in patients aged > 65 years.^23^ In our cohort, the age thresholds for any‐grade and grade ≥2 TRP were 59.5 and 68.5 years, respectively. These findings suggest that younger patients may be more suitable candidates for concurrent ICI‐CCRT.

The phase 3 PACIFIC‐2 trial (NCT0351997), which evaluated durvalumab with CCRT for unresectable stage III NSCLC, did not meet its primary endpoint of improved PFS compared with CRT alone. Further clinical studies, including KEYLYNK‐012,^26^ are necessary to validate the suitability of this treatment approach. Additionally, broader clinical data are needed to define its safety profile, including modifiable predictors, such as dosimetric factors. Beyond the overall elevated TRP risk observed in our cohort, the safety profile of different immunotherapy regimens warrants further consideration. Although this study was not designed to compare treatment subgroups because of the limited sample size, we observed a numerical trend toward a higher incidence of grade ≥3 pneumonitis among patients receiving dual immunotherapy (2 of 11, 18.2%) than among those receiving single‐agent ICI (5 of 58, 8.6%). This trend is consistent with evidence suggesting that dual immune checkpoint blockade, particularly cytotoxic T‐lymphocyte associated protein 4 (CTLA‐4) inhibition combined with PD‐1/PD‐L1 inhibition, is associated with a higher baseline risk of immune‐related pneumonitis, as reported in studies, such as CheckMate 227 in advanced NSCLC.^27^ When intensified immunotherapy is combined with the pro‐inflammatory effects of thoracic radiotherapy, the risk of severe pneumonitis may increase further. This possibility is supported by the CheckMate 012 trial,^28^ which reported a 13% rate of grade ≥3 pneumonitis with concurrent nivolumab, ipilimumab, and thoracic radiotherapy, a rate higher than that typically observed with single‐agent ICI combined with CCRT (e.g., 11.7% in the NICOLAS trial). Therefore, although our data do not support a definitive conclusion regarding comparative safety, they highlight the need for careful risk‐benefit assessment and intensified monitoring when dual ICIs are considered as part of triple‐modality therapy.

The small sample size, cohort heterogeneity, and preliminary nature of the dosimetric analyses limited the ability of this study to detect subtle associations between TRP and other potentially relevant parameters. Nevertheless, the principal parameters evaluated in this study (*V*5, *V*10, *V*15, *V*20, *V*30, and MLD) were selected because they are among the dose‐volume metrics most commonly used in contemporary clinical practice. Our findings suggest that, when thoracic radiotherapy is planned in the context of concurrent ICI administration, accepted MLD constraints may require future revision if these results are corroborated.

## 5 LIMITATIONS

This study has some limitations. First, because concurrent trimodality therapy is not yet a mainstream treatment approach and limited evidence supports^10^, ^11^, ^26^ its routine use, the available sample size was small. Second, because of the small sample size, we did not stratify analyses by ICI type or tumor/histology type. PD‐1 and PD‐L1 inhibitors may be associated with different pneumonitis rates, which may have introduced heterogeneity and bias.^29^ The multi‐center, retrospective design may have further contributed to population heterogeneity and inherent bias. Third, owing to the limited sample size and number of TRP events, we could not separately analyze acute and late‐onset TRP. Moreover, distinguishing immune‐related from radiation‐related pneumonitis remains clinically challenging. Fourth, the AUC values from the ROC analyses were below 0.7, indicating limited discriminative ability and reflecting heterogeneity within the dataset. Finally, variables with subgroups containing markedly few patients could not be reliably included in the logistic regression model. Therefore, these findings require validation in larger, and more homogeneous patient cohorts.

## 6 CONCLUSIONS

In this study, both MLD and age were associated with an increased risk of TRP in patients receiving CCRT and immunotherapy. These findings may help guide future adjustments to lung dose constraints; however, prospective confirmation is necessary.

## CONFLICT OF INTEREST STATEMENT

The authors declare no conflicts of interest.

## ETHICS APPROVAL AND CONSENT TO PARTICIPATE

This study was approved by the Ethics Committee of Hubei Cancer Hospital (Approval Number: LLHBCH2024YN‐047), and obtained informed consent and recognition from all participating sub‐centers.

## Supporting information



Supporting Information

Supporting Information

Supporting Information

Supporting Information

## Data Availability

The raw data supporting the conclusions of this study are available from the corresponding author upon request.
