# Supplementary material for: Dosimetric risk factors for therapy‐related pneumonitis in patients with immunotherapy plus chemotherapy concurrent thoracic radiotherapy
Source: Precis Radiat Oncol. 2026 Nov 3:10.1002/pro6.70102. Online ahead of print. doi: 10.1002/pro6.70102 (PMC13732162; doi:10.1002/pro6.70102)
Supplement: Supplementary file 1 [file PRO6-9999-0-s001.docx]

**Supplement table 3. Dosimetric comparison of different tumor species**

|  | **Esophagus cancer**  (median [IQR]) | **Lung cancer**  (median [IQR]) | P |
| --- | --- | --- | --- |
| N | 25 | 44 |  |
| GTV | 53.000 [33.000, 62.000] | 74.280 [43.775, 134.975] | 0.0144^*^ |
| PTV | 427.558 [390.700, 529.850] | 325.050[218.700, 429.237] | 0.0105^*^ |
| Lung V5 | 50.000 [38.200, 59.000] | 47.730 [40.105, 55.175] | 0.3925 |
| Lung V10 | 36.300 [31.353, 38.996] | 35.300 [31.409, 36.568] | 0.4582 |
| Lung V15 | 27.940 [23.195, 29.168] | 28.645 [25.215, 28.978] | 0.6966 |
| Lung V20 | 18.470 [16.140, 23.230] | 24.125 [19.688, 26.000] | 0.0084^*^ |
| Lung V30 | 9.600 [5.900, 12.550] | 17.000 [13.675, 19.902] | <0.0001^*^ |
| Lung Dmean | 10.900 [8.484, 12.900] | 13.645 [10.878, 15.455] | 0.0013^*^ |

^*^Signifificant (p < 0.05) difference between esophagus cancer and lung cancer.

Abbreviations: N: number; IQR: interquartile range; GTV: gross tumor volume; PTV: planning target volume; Lung V5: the volume of lungs receiving≥5Gy; Lung V10: the volume of lungs receiving≥10Gy; Lung V15: the volume of lungs receiving≥15Gy; Lung V20: the volume of lungs receiving≥20Gy; Lung V30: the volume of lungs receiving≥30Gy; Lung Dmean:.the mean lung dose.
