# Supplementary material for: Dosimetric risk factors for therapy‐related pneumonitis in patients with immunotherapy plus chemotherapy concurrent thoracic radiotherapy
Source: Precis Radiat Oncol. 2026 Nov 3:10.1002/pro6.70102. Online ahead of print. doi: 10.1002/pro6.70102 (PMC13732162; doi:10.1002/pro6.70102)
Supplement: Supplementary file 2 [file PRO6-9999-0-s002.docx]

**Supplement table 2. Dosimetric comparison of three different tumor species**

|  | **Esophagus cancer**  (median [IQR]) | **NSCLC**  (median [IQR]) | **SCLC**  (median [IQR]) | **P** |
| --- | --- | --- | --- | --- |
| N | 25 | 18 | 26 |  |
| GTV | 53.000  [33.000, 62.000] | 105.400  [57.675, 207.425] | 66.250  [38.720, 99.195] | 0.0157^*^ |
| PTV | 427.558  [390.700, 529.850] | 371.800  [274.550, 521.775] | 279.910  [169.900, 415.462] | 0.0081^*^ |
| Lung V5 | 50.000  [38.200, 59.000] | 50.620  [40.240, 56.195] | 46.330  [40.938, 54.443] | 0.6937 |
| Lung V10 | 36.300  [31.353, 38.996] | 39.500  [28.553, 39.547] | 34.950  [31.369, 36.522] | 0.5225 |
| Lung V15 | 27.940  [23.195, 29.168] | 29.850  [22.657, 30.722] | 28.235  [25.530, 29.228] | 0.7359 |
| Lung V20 | 18.470  [16.140, 23.230] | 24.325  [16.087, 26.150] | 23.600  [20.117, 25.970] | 0.0289^*^ |
| Lung V30 | 9.600 [  5.900, 12.550] | 18.285  [12.150, 19.758] | 16.170  [14.000, 19.825] | <0.0001^*^ |
| Lung Dmean | 10.900  [8.484, 12.900] | 14.925  [10.625, 15.635] | 12.998  [11.038, 14.697] | 0.0052^*^ |

^*^Significant difference (P < 0.05) among the groups.

Abbreviations: N: number; NSCLC: non-small cell lung cancer; SCLC: small cell lung cancer; IQR: interquartile range; GTV: gross tumor volume; PTV: planning target volume; Lung V5: the volume of lungs receiving≥5Gy; Lung V10: the volume of lungs receiving≥10Gy; Lung V15: the volume of lungs receiving≥15Gy; Lung V20: the volume of lungs receiving≥20Gy; Lung V30: the volume of lungs receiving≥30Gy; Lung Dmean:.the mean lung dose.
