# Supplementary material for: Dosimetric risk factors for therapy‐related pneumonitis in patients with immunotherapy plus chemotherapy concurrent thoracic radiotherapy
Source: Precis Radiat Oncol. 2026 Nov 3:10.1002/pro6.70102. Online ahead of print. doi: 10.1002/pro6.70102 (PMC13732162; doi:10.1002/pro6.70102)
Supplement: Supplementary file 3 [file PRO6-9999-0-s003.docx]

**Supplement table 4. Comparison of radiation dosimetry in different grades of TRP**

|  | **Grade0 TRP** (median [IQR]) | **Grade1 TRP** (median [IQR]) | **Grade≥2 TRP** (median [IQR]) | **P** |
| --- | --- | --- | --- | --- |
| N | 30 | 11 | 28 |  |
| GTV | 57.159  [32.925, 69.650] | 76.700  [51.400, 121.000] | 70.532  [42.038, 130.325] | 0.1239 |
| PTV | 297.000  [195.493, 426.525] | 425.000  [379.750, 478.600] | 418.600  [304.780, 493.650] | 0.0773 |
| Lung_V5 | 46.070  [36.390, 56.823] | 49.600  [40.820, 57.490] | 50.500  [42.198, 54.850] | 0.7446 |
| Lung_V10 | 33.815  [28.577, 35.809] | 36.300  [30.765, 40.944] | 38.900  [33.237, 39.239] | 0.3030 |
| Lung_V15 | 27.720  [22.360, 27.676] | 27.900  [23.879, 31.212] | 30.350  [25.992, 30.670] | 0.1333 |
| Lung_V20 | 20.705  [17.005, 24.343] | 24.000  [15.880, 25.900] | 24.175  [18.352, 26.135] | 0.1217 |
| Lung_V30 | 12.625  [9.600, 15.908] | 15.100  [8.200, 18.760] | 16.390  [11.625, 20.035] | 0.1561 |
| Lung_Dmean | 11.239  [9.703, 12.975] | 13.400  [8.762, 14.995] | 13.840  [10.892, 15.593] | 0.0246^*^ |

^*^Significant difference (P < 0.05) among the groups.

Abbreviations: TRP: treatment-related pneumonitis; IQR: interquartile range; N: number; GTV: gross tumor volume; PTV: planning target volume; Lung V5: the volume of lungs receiving≥5Gy; Lung V10: the volume of lungs receiving≥10Gy; Lung V15: the volume of lungs receiving≥15Gy; Lung V20: the volume of lungs receiving≥20Gy; Lung V30: the volume of lungs receiving≥30Gy; Lung Dmean:.the mean lung dose.
