# Supplementary material for: Dosimetric risk factors for therapy‐related pneumonitis in patients with immunotherapy plus chemotherapy concurrent thoracic radiotherapy
Source: Precis Radiat Oncol. 2026 Nov 3:10.1002/pro6.70102. Online ahead of print. doi: 10.1002/pro6.70102 (PMC13732162; doi:10.1002/pro6.70102)
Supplement: Supplementary file 4 [file PRO6-9999-0-s004.docx]

**Supplement table 1. Comparison of the incidence of TRP between different tumors**

|  | **NSCLC(%)** | **SCLC(%)** | **Esophagus cancer(%)** | **P** |
| --- | --- | --- | --- | --- |
| **N** | 18 | 26 | 25 |  |
| **TRP** |  |  |  | 0.292 |
| No | 5 (27.8) | 13 (50.0) | 12 (48.0) |  |
| Yes | 13 (72.2) | 13 (50.0) | 13 (52.0) |  |
| **Grade3-5 TRP** |  |  |  | 0.566 |
| No | 15 (83.3) | 24 (92.3) | 23 (92.0) |  |
| Yes | 3 (16.7) | 2 (7.7) | 2 (8.0) |  |

Abbreviations: N: number; NSCLC: non-small cell lung cancer; TRP: treatment-related pneumonitis; SCLC: small cell lung cancer; EC: esophageal cancer
